# Differentiation Potential of Human Wharton's Jelly-Derived Mesenchymal Stem Cells and Paracrine Signaling Interaction Contribute to Improve the In Vitro Maturation of Mouse Cumulus Oocyte Complexes

**DOI:** 10.1155/2018/7609284

**Published:** 2018-10-11

**Authors:** Martin Maldonado, Tianhua Huang, Jianying Chen, Ying Zhong

**Affiliations:** Reproductive Medicine & Genetics, Chengdu Jinjiang Hospital for Maternal & Child Health Care, Chengdu 610066, China

## Abstract

In vitro maturation (IVM) in cumulus oocyte complexes (COCs) can be improved by the presence of human Wharton's jelly-derived MSCs (hWJ-MSCs), under specific culture conditions. COCs were cultured in twelve different culture systems, composed of four stock media, stock media conditioned with hWJ-MSCs, and stock media in which the oocytes were indirectly cocultured with the hWJ-MSCs. The rates of maturation to meiosis II were compared among the groups. G2-PLUS and coculture with DMEM-F12 were the most efficient systems for the maturation of COCs. The fertilization rate and rate of development to the blastocyst stage were compared between these two groups. Moreover, hWJ-MSC-conditioned media showed no benefits for the COC-IVM. The analysis of OCT4 expression of hWJ-MSCs in G1-PLUS, TYH, and G2-PLUS showed a downregulation of OCT4 by 25.9, 24.7, and 6.6%, respectively, compared to that in hWJ-MSCs cultured in DMEM-F12. Finally, we have demonstrated that two prerequisites appeared to be necessary for the hWJ-MSCs to improve the IVM of COCs: hWJ-MSCs' differentiation potential and the presence of coordinated paracrine interaction between the stem cells and COCs. Under the appropriate conditions, the paracrine factors produced in the coculture system with DMEM-F12 may help to develop synthetic media for successful in vitro culture of COCs.

## 1. Background

In vitro maturation (IVM) in cumulus oocyte complexes (COCs) can be improved by the presence of human Wharton's jelly-derived MSCs (hWJ-MSCs), under specific culture conditions.

Mesenchymal stem cells (MSCs) are attractive candidates for cell-based therapeutic strategies, primarily due to their intrinsic ability to self-renew and undergo multipotential differentiation and because they are amenable to genetic manipulation [1–3].

In a coculture, MSCs not only provide a target cell source with multipotent differentiation capacity but can also act as assisting cells to promote tissue homeostasis, metabolism, growth, and repair [4]. Their incorporation into coculture systems seems to be important for creating complex tissues or organs by cell-to-cell contact or/and through the delivery of soluble factors to the target cells.

Among the different types of MSCs, human Wharton's jelly-derived MSCs (hWJ-MSCs) appear to offer the best clinical advantages due to their unique beneficial characteristics [5]. hWJ-MSCs reprogram resident cells to favor tissue regeneration, attenuate wound inflammation, and inhibit fibrosis [6, 7]. They interact with host cells and influence the stem cell niche through differentiation and/or paracrine signaling mechanisms [6, 8, 9]. They are poor antigen-presenting cells; do not express MHC class II or the costimulatory molecules CD40, CD40L, CD80, and CD86 [10, 11]; and are not prone to undergo malignant transformation [12, 13].

Many attempts have been made to grow immature oocytes *in vitro* [14–16]. The culture conditions used for *in vitro* maturation (IVM), which include various media types and hormone supplements, as well as the presence of cumulus cells, can significantly influence maturation rates and subsequent embryo development [14, 17–19].

Furthermore, during ovarian follicular development, several growth factors and cytokines act as intraovarian regulators *in vivo*, and in the follicle, the action of gonadotrophins is modulated by locally produced paracrine and autocrine growth factors [20, 21].

Scientific evidence indicates that the secretion of trophic, soluble, or immunomodulatory factors, known as paracrine signals, may represent the most important underlying mechanism of MSC effects [9, 22, 23].

Given the appropriate stimuli and local environment, MSCs not only can develop into various cell types in vitro and regenerate tissues in vivo, but they can also secrete a variety of cytokines and growth factors, such as MCP-1, VEGF-A, EGF, FGF-2, IL-6, LIF, and TGF-*β* [2, 24].

Since cytokines and growth factors are known to stimulate meiotic progress and the processes associated with IVM, the aim of the present study was to determine whether IVM in cumulus oocyte complexes (COCs) could be improved by the presence of hWJ-MSCs through nonspecific release of cytokines and soluble factors using conditioned medium or by paracrine signaling from hWJ-MSCs using indirect coculture conditions. However, the success of IVM of COCs in coculture also depends on the choice of a suitable medium to allow oocyte maturation and the survival and potency of the hWJ-MSCs. In this work, we have opted to use DMEM-F12, a defined combination of nutrients, growth factors, and hormones that supports stemness and the differentiation potential of MSCs; two commercially available culture media for culture of human embryos from after fertilization until the 8-cell stage (G1-PLUS); a medium for culture of human embryos from the 8-cell stage on day three until the blastocyst stage (G2-PLUS); and TYH, first reported as a medium for in vitro fertilization (IVF) of mouse eggs with epididymal spermatozoa, by Yoda et al. [25] and Toyoda et al. [26] in 1971.

## 2. Methods

### 2.1. Animals

Male and female Kunming mice were maintained in a specific-pathogen-free animal facility in individual ventilated cages and housed at 23°C under a 12-hour dark/light cycle. Water and food were given *ad libitum.* All animal work was performed using protocols approved by the Institutional Review Board of the Chengdu Jinjiang Maternity and Child Health Hospital.

The health conditions of mice were monitored daily. Prior to the experimental endpoint, mice experienced minimal pain or stress during routine handling and hormone administration.

No ill or dead mice were observed prior to the experimental endpoint. Mice were euthanized by cervical dislocation.

### 2.2. Oocyte Handling

Mouse COCs were retrieved from 6- to 8-week-old Kunming mice (Chengdu Da Suo Biology and Technology Company) by ovary puncture with a 28 G sterile needle under a stereomicroscope. COCs were isolated 48 hours after an intraperitoneal injection of 10 IU of pregnant mare serum gonadotropin (PMSG, Sigma Chemical Co., St. Louis, MO). G-MOPS™ PLUS was used for handling and manipulation of the oocytes outside the incubator. Groups of oocytes were separated and cultured in 12 different culture systems and divided into 3 experimental stages. The first experimental stage included the following stock media preparations: (a) DMEM-F12 (Gibco; Thermo Fisher Scientific Inc., Waltham, MA, USA), (b) G1-PLUS (Vitrolife), (c) G2-PLUS (Vitrolife), and (d) TYH medium (Table S1). The second experimental stage included the following types of hWJ-MSC-conditioned media: (e) conditioned-DMEM-F12, (f) conditioned-G1-PLUS, (g) conditioned-G2-PLUS, and (h) conditioned-TYH. The third experimental stage included the following types of coculture with hWJ-MSCs: (i) coculture in DMEM-F12, (j) coculture in G1-PLUS, (k) coculture in G2-PLUS, and (l) coculture in TYH. The maturation status was evaluated after 24 hours of in vitro culture at 37°C in 6% CO_2_. Oocytes with polar bodies were considered mature MII oocytes.

For all the experimental assays in this study, DMEM-F12 was supplemented with 10% fetal bovine serum (FBS, Thermo Fisher Scientific).

### 2.3. Isolation and Culture of hWJ-MSCs

Protocols for sampling the human umbilical cord were approved by the Institutional Review Board of Chengdu Jinjiang Hospital for Maternal & Child Health Care, according to the principles expressed in the Declaration of Helsinki. All participants provided their written consent to participate in this study. Human umbilical cords were obtained from consenting patients delivering full-term infants by cesarean section. The procedures for culture of hWJ-MSCs were consistent with those of Huang et al., with minor modifications [27]. Briefly, the arteries and veins of each tissue were mechanically removed, and the subamniotic region of the Wharton jelly was transferred to a sterile container and diced into small fragments. The explants were transferred into 100 mm plates with fresh growth media containing DMEM-F12 (Gibco)/FBS. The cells were left undisturbed for 7 days in a 37° humidified incubator with 5% CO_2_ to allow migration of cells from the explants. Thereafter, the tissue was removed, and the hWJ-MSCs were evenly redistributed by digesting them with TrypLE (Gibco). Fresh media (4-5 ml) were added every 2/3 days. Then, the cells (±90% confluence) were digested and replated at a 1 : 5 ratio.

We have previously assessed the phenotypic properties of hWJ-MSCs based on the immune response-related surface markers CD80, CD86, CD40, CD40L, HLA-1, and HLA-DR [12].

### 2.4. Preparation of Conditioned Media

hWJ-MSCs (80% confluence) were cultured for 24 hours with each one of the following media types: (a) DMEM-F12, (b) G1-PLUS, (c) G2-PLUS, or (d) TYH. Thereafter, the media were collected and immediately used for COC IVM.

### 2.5. Indirect Coculture of COCs with hWJ-MSCs

hWJ-MSCs were plated in 24.5 mm, 3 *μ*m Transwel culture plates (Corning™, Inc.). COCs were allocated on the permeable polycarbonate membrane and coincubated with the hWJ-MSCs for 24 hours in the following media conditions: (a) hWJ-MSC-COCs-DMEM-F12, (b) hWJ-MSC-COCs-G1-PLUS, (c) hWJ-MSC-COCs-G2-PLUS, or (d) hWJ-MSC-COCs-TYH.

### 2.6. Quantitative Real-Time Polymerase Chain Reaction (qPCR) Assay

The OCT4 expression of hWJ-MSCs in the 4 coculture systems were analyzed by qPCR. The cells were homogenized, and the RNA was extracted with the TaKaRa MiniBEST Universal RNA Extraction Kit (TaKaRa Bio Inc.). The total RNA concentration and O.D. were assayed by UV spectrophotometry. Genomic DNA elimination was performed with TaKaRa RR047Q. qPCR was performed with 200 ng of target cDNA; OCT4 (forward, GTGTTCAGCCAAAAGACCATCT; reverse, GGCCTGCATGAGGGTTTCT) and *β*-actin (Table S2), which was used as a reference gene (forward, CCTCATGAAGATCCTCACCGA; reverse, TTGCCAATGGTGATGACCTGG), were evaluated using relative quantification with SYBR Premix Ex Taq™ (Takara RR420Q) using an ABI 7500 (Applied Biosystems, Foster City, CA). The data analyzed was collected from 3 different experiments and the samples were run in triplicate.

### 2.7. IVM and IVF

IVM, with standard and conditioned media, was performed in 50 *μ*l microdrops of medium containing 10 COCs per microdrop, under mineral oil submersion for 24 hours at 37°C with 6% CO_2_. For coculture, groups of ±50 COCs were cultured with hWJ-MSCs in the various combinations of media, as explained in Section 2.5. The oocytes were monitored for IVM by confocal microscopy at 0 and 24 hours.

IVF then was performed as follows. Briefly, epididymis tissues from Kunming mice were isolated and subjected to small cuts with scissors. The growth medium (1 ml of G2-PLUS or DMEM-F12) was added, and the mixture was incubated for 7 min at 37°C. Then, the supernatant was centrifuged at 650 ×g for 5 min and discarded. The remaining pellet was resuspended by pipetting with 1 ml of medium and incubated at 37°C for 1 hour (capacitation). A second centrifugation was performed, and 700 *μ*l of the 800 *μ*l of supernatant was discarded. The remaining volume contained the activated spermatozoa. Groups of oocytes (*n* = 20) were inseminated in 80 *μ*l microdrops of the activated spermatozoa in G2-PLUS medium (under equilibrated mineral oil at 37°C in air with 6% CO_2_). For IVF in coculture conditions, groups of oocytes (*n* = 20) were inseminated in 80 *μ*l microdrops previously cultured with hWJ-MSCs in DMEM-F12 medium. At the time of insemination, the DMEM-F12 medium was replaced with the activated spermatozoa in DMEM-F12 medium. A portion of the oocytes from each treatment was incubated without spermatozoa to evaluate the incidence of spontaneous activation (data not shown). Six hours after insemination, the oocytes were gently cleaned by pipetting, and presumptive zygotes obtained from COCs were separately cultured *in vitro* for 5 days in G2-PLUS medium or cocultured with hWJ-MSCs in DMEM-F12 medium (37°C, in air with 6% CO_2_). The proportion of the embryos at each stage was recorded at 24 hours, 48 hours, and after 5 days of *in vitro* culture.

### 2.8. Statistical Analysis

Statistical analysis was performed using Microsoft Office Excel 2010. The oocyte maturation, fertilization, and embryonic development rates were calculated using the Pearson chi-squared test. The qPCR data was analyzed with the two-tailed paired Student's *t*-test. A *P* value < 0.05 was considered to indicate statistical significance.

## 3. Results

### 3.1. IVM and IVF of COCs

A total of 2422 immature oocytes met the eligibility criteria for this study. The developmental competency of COCs is listed in Table 1.

Among the 12 culture systems analyzed in this work, G2-PLUS and coculture-DMEM-F12 were the most effective media conditions for IVM. Both showed no significant differences in their ability to improve oocyte maturation and were statistically different from the rest of the systems.

**Figure 1:**
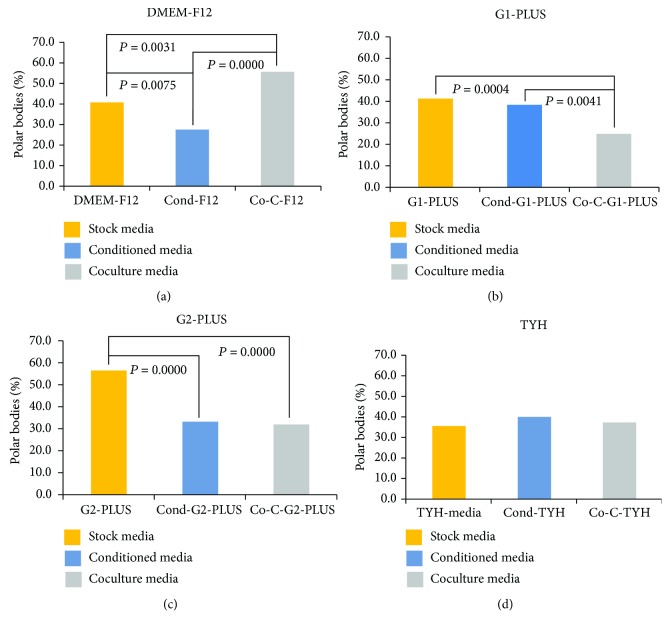
Comparison of IVM for COCs cultured in the same media: DMEM-F12 (a), G1-PLUS (b), G2-PLUS (c), and TYH (d). The data were analyzed by the chi-squared test. A value of *P* < 0.05 was considered statistically significant.

When the groups were compared according to media type (Figure 1), we noticed that conditioning DMEM-F12 (Figure 1(a)) and G2-PLUS (Figure 1(c)) with hWJ-MSCs yielded negative outcomes compared to those from stock media, suggesting that the release of unspecific soluble factors from hWJ-MSCs may have an adverse effect on the oocyte maturation. In conditioned-G1-PLUS (Figure 1(b)), IVM was not significantly different from the stock media. Moreover, no improvement was noted in conditioned-TYH culture (Figure 1(d)).

On the other hand, the coculture system in G1- and G2-PLUS media appeared to have detrimental effects on IVM (Figures 1(b) and 1(c)) compared to the effects of culture in stock media. Coculture in TYH showed no significant differences with stock media conditions (Figure 1(d)). However, the IVM of COCs in coculture with hWJ-MSC-DMEM-F12 was significantly improved compared to IVM in stock DMEM-F12 (Figure 1(a)).

We also analyzed the fertilization and developmental rates of oocytes cultured in G2-PLUS and cocultured with DMEM-F12 media. Although we noticed a modest improvement in the rate of development to the blastocyst stage in the coculture with DMEM-F12, the result was not significant at *P* < 0.05 (Table 1). Oocytes, either cultured in G2-PLUS or cocultured in DMEM-F12 media, cleaved and likely did not experience developmental arrest at the pronuclear stage.

### 3.2. Analysis of OCT4 Expression

**Figure 2:**
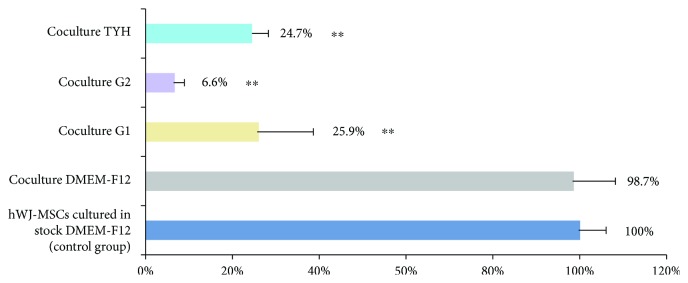
qPCR analysis of OCT4 in hWJ-MSCs cultured under the 4 coculture conditions. In G1-PLUS, TYH, and G2-PLUS, OCT4 expression was downregulated to 25.9, 24.7, and 6.6%, respectively, compared to the OCT4 level in hWJ-MSCs cultured in DMEM-F12. The expression of OCT4 from hWJ-MSCs was not affected by the presence of COCs when cultured in DMEM-F12 (control group). The data was analyzed with the two-tailed paired Student's *t*-test. A *P* value < 0.05 was considered to indicate statistical significance. ^∗∗^(*P* < 0.01) is the statistical significance compared with coculture-DMEM-F12.

The POU transcription factor OCT4 (Table S2), encoded by POU5F1, is critical for sustaining the self-renewal capacity of adult somatic stem cells [28, 29] and is considered a master regulator of pluripotency that controls lineage commitment [30]. We analyzed OCT4 expression of hWJ-MSCs, by qPCR in the 4 different stock media with the aim of verifying whether the specific media affected hWJ-MSC potency. Our data confirmed that the expression of OCT4 in hWJ-MSCs cultured on G1-PLUS, TYH, and G2-PLUS was downregulated by 25.9, 24.7, and 6.6%, respectively, compared to that in hWJ-MSCs cultured in DMEM-F12 (Figure 2). We also confirmed that the expression of OCT4 from hWJ-MSCs was not affected by the presence of COCs when cultured in DMEM-F12.

## 4. Discussion

Culture conditions, including the formulation of the base medium, supplementations, and the in vitro physical environment (such as the oxygen tension and presence of cumulus cells), all influence multiple events that are crucial to oocyte maturation and subsequent embryonic development [31, 32].

The culture media types have been shown to modulate not only the metabolism of oocytes [33] but also the maturation to MII, the kinetics of cell cycle progression, and spindle/chromatin organization [34, 35].

Scientific evidence suggests that gonadotropins, epidermal growth factor, and essential and nonessential amino acids improve the maturation of porcine oocytes [36, 37]. Human menopausal gonadotropin, pregnant mare serum gonadotropin, follicle-stimulating hormone (FSH), and luteinizing hormone, pyruvic acid, estradiol (E2), recombinant FSH, insulin, transferrin, and selenium have been included in the IVM media for human oocyte maturation [38–40]. Undefined protein sources, such as fetal bovine serum, human maternal serum, synthetic human serum, and human follicular and peritoneal fluid have also been used to supplement maturation media [41–43].

Although taurine and calcium lactates have been suggested as necessary components for IVM of mouse COCs [30], the two most efficient culture media (G2-PLUS and DMEM-F12) tested in this work lacked these compounds.

It is evident that there is no consensus on the adequate supplementation media for oocyte maturation as the postulated compounds and combinations stated above have yielded suboptimal results, thus reinforcing the idea that the in vitro culture conditions lack some crucial components.

One of the shortcomings in the type of culture system used to date has been the absence of somatic cell support.

The role of stem cells in coculture reaches beyond that of simply providing a favorable cell source with multipotent differentiation capacity. Whether serving in target or assisting roles, stem cells are pivotal in tissue growth, metabolism, maturation, and repair [44, 45].

Many kinds of exogenous growth factors are secreted by MSCs, some of which are important components of the follicle developmental microenvironment for appropriate “oocyte capacitation” or oocyte cytoplasmic development prior to maturation [20].

The present work supported the idea that remote cell signaling from hWJ-MSCs positively affects meiotic progression of COCs maintained in the same microenvironment with DMEM-F12, resulting in a defined combination of nutrients, growth factors, and hormones that supports oocyte maturation and the differentiation potential of MSCs.

Our data also suggest that the paracrine factors produced by hWJ-MSCs in DMEM-F12 helped cocultured COCs to reach levels of nuclear and cytoplasmic maturation similar to those seen in culture with G2-PLUS, a blastocyst culture medium with superior efficiency for IVM of immature oocytes [30]. However, the specific compounds produced by the hWJ-MSCs, such as cytokines and soluble and growth factors that might be involved in the regulation of oocyte maturation, need to be identified.

In addition, the beneficial effect of coculture did not require any direct contact between the hWJ-MSCs and COCs during IVM and IVF, as previously reported for bovine and mouse oocytes [46, 47], supporting the idea that cell-cell interaction occurs via soluble factors triggered by remote cell signaling.

The scientific evidence suggests that some of the positive effects of soluble factors secreted by hWJ-MSCs might be due to antioxidant properties, especially since it is now recognized that exogenous antioxidants improve maturation *in vitro* in the cat [48] and specifically support the MI to MII transition in pig oocytes [49].

The above statements appear to be consistent with our results showing that the hWJ-MSC-conditioned medium had no positive effect on the IVM of COCs and suggest that coordinated paracrine signaling communication is required between the assisting (hWJ-MSCs) and the target cells (COCs).

We evaluated the fertilization rate and extended culture up to the blastocyst stage to detect the maturation and developmental competence of the oocytes, which should be better indicators than molecular markers or detailed morphological studies.

We also verified that oocytes, either cultured in G2-PLUS or cocultured in DMEM-F12 media, cleaved and likely did not experience developmental arrest at the pronuclear stage. Nonetheless, further studies should be conducted to ascertain the implantation and pregnancy potential of these embryos, and detailed analysis of their chromosomal status or genomic imprinting pattern should be performed.

OCT4 is a critical transcription factor for regulating the self-renewal and differentiation properties of ESCs [50] and its downregulation induces loss of pluripotency and dedifferentiation to the trophectoderm [51].

hWJ-MSCs constitutively express the early embryonic transcription factors Nanog, OCT4, Sox-2, Rex-1, and LIN28, indicating their multipotency and high self-renewal capacity [52, 53].

Moreover, the expression of the pluripotency genes, OCT4, Nanog, and Sox-2, was also reported for WJ-MSCs [52, 54, 55], although it was much lower than that in ESC [53]. Modest expression of pluripotency genes might explain why WJ-MSCs are not tumorigenic.

In our work, we have opted to analyze the pluripotency gene OCT4 as a tool for the identification of cell differentiation since up- or downregulation of OCT4 indicates loss of differentiation potential and stemness regardless of the expression of the other markers of pluripotency expressed in these cells.

We have already suggested that specific MSC media compositions are necessary for the hWJ-MSCs to positively interact with COCs and improve IVM, which is also correlated with the expression of OCT4 and consequently with the differentiation potential of the hWJ-MSCs. However, further investigation with different commercially available media formulations that support MSC culture will be required to support this idea.

Importantly, cell-cell interactions are seldom unidirectional, with both cell populations being affected in a coculture system. However, the rate at which COCs were cocultured with hWJ-MSCs in this study demonstrated that oocytes had no effect on the OCT4 expression of hWJ-MSCs; thus, the potency of the stem cells remained unaffected.

## 5. Conclusion

Our findings support the concept of cross-talk between hWJ-MSCs and COCs in indirect coculture. We have demonstrated that two prerequisites appear to be necessary for hWJ-MSCs to improve COC IVM: stem cell differentiation potential and paracrine signaling interaction with the COCs. The paracrine factors produced by hWJ-MSCs in the coculture system with the DMEM-F12 medium may guide the development of synthetic media for successful in vitro culture of COCs.

## Figures and Tables

**Table 1:** Developmental competency of COCs among the different culture systems.

Culture media	Total number of COCs	Polar bodies (number)	Polar bodies (%)	Number of cleaved oocytes (%)	Number of blastocyst stage (%)
DMEM-F12	199	81	40.7		
G1-PLUS	204	85	41.6		
G2-PLUS	199	115	57.8^∗^	61 (53.04)	7 (11.48)
TYH	198	71	35.9		
Conditioned-DMEM-F12	200	56	28		
Conditioned-G1-PLUS	206	79	38.4		
Conditioned-G2-PLUS	204	69	33.8		
Conditioned-TYH	202	81	40.1		
Coculture-DMEM-F12	205	115	56.1^∗^	58 (50.43)	8 (13.79)
Coculture-G1-PLUS	203	51	25.1		
Coculture-G2-PLUS	206	66	32		
Coculture-TYH	196	74	37.7		

Oocytes with polar bodies were considered mature MII oocytes. The fertilization rate and rate of development to the blastocyst stage were compared between G2-PLUS and coculture-DMEM-F12. ^∗^Both showed no significant differences in their ability to improve oocyte maturation and were statistically different from the rest of the systems. The data were analyzed by the chi-squared test. A value of *P* < 0.05 was considered statistically significant.

## Data Availability

All data generated or analyzed during this study are included in this published article (and its supplementary information files).

## Supplementary Materials

Supplementary Materials Table S1: TYH medium formulation. Table S2: list of primers.
