# Supplementary material for: Differentiation Potential of Human Wharton's Jelly-Derived Mesenchymal Stem Cells and Paracrine Signaling Interaction Contribute to Improve the In Vitro Maturation of Mouse Cumulus Oocyte Complexes
Source: Stem Cells Int. 2018 Oct 11;2018:7609284. doi: 10.1155/2018/7609284 (PMC6201336; doi:10.1155/2018/7609284)
Supplement: Supplementary Materials — Table S1: TYH medium formulation. Table S2: list of primers. [file 7609284.f1.pdf]

Table S1

TYH Medium	(mg / 100ml)
BSA	300
$CaCl_2 \cdot 2H_2O$	25.1
Glucose	100.2
KCl	35.6
KH_2PO_4	16.2
$MgSO_4 \cdot 7H_2O$	29.3
NaCl	697.6
$NaHCO_3$	210.6
Phenol red	several drops
Sodium pyruvate($C_3H_3NaO_3$)	11

Table S2

Oligo name	Forward sequence (5’ to 3’)	Reverse sequence (5’ to 3’)	Product size (bp)	Accession number[a]
ACTB	CCTCATGAAGATCCTCACCGA	TTGCCAATGGTGATGACCTGG	192	XM_0067157 4
POUF51	GTGTTCAGCCAAAAGACCATCT	GGCCTGCATGAGGGTTTCT	156	NM_0011595 2
